# Extended SIR Prediction of the Epidemics Trend of COVID-19 in Italy and Compared With Hunan, China

**DOI:** 10.3389/fmed.2020.00169

**Published:** 2020-05-06

**Authors:** Jia Wangping, Han Ke, Song Yang, Cao Wenzhe, Wang Shengshu, Yang Shanshan, Wang Jianwei, Kou Fuyin, Tai Penggang, Li Jing, Liu Miao, He Yao

**Affiliations:** ^1^Beijing Key Laboratory of Aging and Geriatrics, National Clinical Research Center for Geriatrics Diseases, Second Medical Center of Chinese PLA General Hospital, Institute of Geriatrics, Beijing, China; ^2^Department of Military Medical Technology Support, School of Non-commissioned Officer, Army Medical University, Shijiazhuang, China

**Keywords:** COVID-19, coronavirus, Italy, prediction, epidemics trend

## Abstract

**Background:** Coronavirus Disease 2019 (COVID-19) is currently a global public health threat. Outside of China, Italy is one of the countries suffering the most with the COVID-19 epidemic. It is important to predict the epidemic trend of the COVID-19 epidemic in Italy to help develop public health strategies.

**Methods:** We used time-series data of COVID-19 from Jan 22 2020 to Apr 02 2020. An infectious disease dynamic extended susceptible-infected-removed (eSIR) model, which covers the effects of different intervention measures in dissimilar periods, was applied to estimate the epidemic trend in Italy. The basic reproductive number was estimated using Markov Chain Monte Carlo methods and presented using the resulting posterior mean and 95% credible interval (CI). Hunan, with a similar total population number to Italy, was used as a comparative item.

**Results:** In the eSIR model, we estimated that the mean of basic reproductive number for COVID-19 was 4.34 (95% CI, 3.04–6.00) in Italy and 3.16 (95% CI, 1.73–5.25) in Hunan. There would be a total of 182 051 infected cases (95%CI:116 114–274 378) under the current country blockade and the endpoint would be Aug 05 in Italy.

**Conclusion:** Italy's current strict measures can efficaciously prevent the further spread of COVID-19 and should be maintained. Necessary strict public health measures should be implemented as soon as possible in other European countries with a high number of COVID-19 cases. The most effective strategy needs to be confirmed in further studies.

## Introduction

Corona Virus Disease 2019 (COVID-19) started in Wuhan, China, in December and quickly spread throughout China and to many countries and regions in the world (1–3). The COVID-19 outbreak was declared a pandemic by the World Health Organization (WHO) on March 11. It is currently a global public health threat and more than 100 countries including Italy, Iran, the United States, South Korea, and Japan are suffering from COVID-19. Outside of China, Italy is one of the countries suffering the most with the COVID-19 epidemic. As of April 02, the cumulative number of confirmed cases in Italy reached 115,242, ranking second in the world, with the total confirmed deaths at 13,915, which has become one of the highest among the major epidemic countries. However, few studies have assessed the epidemic status in Italy (4, 5).

Global public health measures are required to cope with the rapid spread of the epidemic. China has taken precise and differentiated strategies, including self-quarantine of residents in Wuhan and other areas and community-based prevention and control. These measures have played an important role in preventing and controlling the epidemic. Previous studies have shown that due to the isolation of Wuhan, the overall epidemiological progress in mainland China has been delayed by 3–5 days and the number of internationally transmitted cases has been reduced by nearly 80% (6). Italy detected the first two cases of imported COVID-19 on Jan 31. After that, Italy was the first country to declare a state of emergency. Since then, various measures have been implemented to control the spread of COVID-19. It is vital to evaluate the role of Italian quarantine measures for decision-making.

Mathematical modeling is helpful to predict the possibility and severity of disease outbreak and provide key information for determining the type and intensity of disease intervention. The SIR model and its modifications such as SEIR model have been widely applied to the current outbreak of COVID-19. Tang et al. estimated the infectivity of COVID-19 based on a classical susceptible-exposed-infected-removed (SEIR) epidemiological model (7). Wu et al. proposed an extended SEIR model to forecast the spread of 2019-nCoV both within and outside of mainland China (3). However, these studies assumed that the exposed population were not infectious, which may be not suitable in COVID-19. Yang Z et al. predicted that China's epidemic will peak in late February and end in late April by a combination of SEIR model and a machine-learning artificial intelligence (AI) approach (8). However, this study and the above studies did not consider the phase-adjusted preventive measures and time-varying parameters, which may affect the accuracy of predictions.

We adopted extended susceptible-infected-removed (eSIR) model (9), which covers the effects of different epidemic prevention measures in different periods and helps to achieve the following specific objectives:

AIM 1: Compare the epidemic development of COVID-19 in Italy with provinces with a similar total population to China.

AIM 2: Predict the epidemiological trend of COVID-19 in Italy via a modified and calibrated model.

## Methods

### Data Sources

In this study, we used the publicly available dataset of COVID-19 provided by the Johns Hopkins University (10). This dataset includes many countries' daily count of confirmed cases, recovered cases, and deaths. As time-series data, it is available from 22 January 2020. We also gathered and cross-checked data in DXY.cn (11), a website providing real-time data of COVID-19.

These data are collected through public health authorities' announcements and are directly reported public and unidentified patient data, so ethical approval is not required.

### Prediction Models

The reproduction number, R0, reflects the transmissibility of a virus spreading under no control, representing the average number of new infections generated by each infected person (12). COVID-19 is likely to decline and eventually disappear if R0 ≤ 1.To estimate trends and calculate the R0, we used an extended SIR model (eSIR model) with a time-varying transmission rate (9). The eSIR model uses a daily-updated time series of infected and removed (recovered and death) proportions as input data. Accordingly, the input data for Italy come from Feb 21 to Apr 02 and the input data for Hunan come from Jan 30 to March 14. By incorporating time-varying transmissions rates, the eSIR model is one extension to the standard SIR model for infectious disease.

#### Standard SIR Epidemiological Model

The standard SIR epidemiological model has three components: susceptible, infected, and removed (including the recovery and dead). The infected cases refer to the current confirmed cases; the removed cases refer to the recovered and death cases.


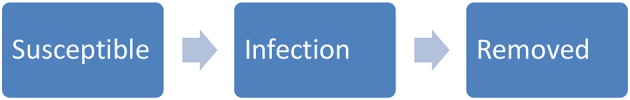


$$\begin{array}{l} \frac{d\theta_{dt}^{S}}{dt}=-\beta \theta_{t}^{S}\theta_{t}^{I},\frac{d\theta_{t}^{I}}{dt}=\beta \theta_{t}^{S}\theta_{t}^{I}-\gamma \theta_{t}^{I},\frac{d\theta_{t}^{R}}{dt}=\;\gamma \theta_{t}^{I} \end{array}$$

Let $Y_{t}^{I}$ and $Y_{t}^{R}$ be the proportions of infection and removed state at time *t*. We assume $Y_{t}^{I}$ and $Y_{t}^{R}$ follows a Beta-Dirichlet stat-space model(BDSSM), consisting of two observation processes:

$$\begin{array}{l} \;Y_{t}^{I}|\theta_{t},\tau \sim Beta\left(\lambda^{I}\theta_{t}^{I},\lambda^{I}\left(1-\theta_{t}^{I}\right)\right),\\ \;Y_{t}^{R}|\theta_{t},\tau \sim Beta\left(\lambda^{R}\theta_{t}^{R},\lambda^{R}\left(1-\theta_{t}^{R}\right)\right), \end{array}$$

And the latent process

$$\begin{array}{l} \;\theta_{t}|\theta_{t-1},\tau \sim \text{Dirichlet}\left(\kappa f\left(\theta_{t-\;1},\beta ,\gamma \right)\right), \end{array}$$

where θ_*t*_ = ${\left(\theta_{t}^{S},\theta_{t}^{I},\theta_{t}^{R}\right)}^{⊤}$ is the vector of the underlying prevalence of the susceptible, infectious, and removed populations, and τ = ${\left(\beta ,\gamma ,\theta_{t}^{⊤},\lambda ,\kappa \right)}^{⊤}$ with λ^*I*^, λ^*R*^ and κ being parameters controlling respective variances for the observation and latent processes.

*f*(.) is be the solution to:

$$\begin{array}{l} \frac{d\theta_{t}^{S}}{dt}=-\beta \theta_{t}^{S}\theta_{t}^{I},\frac{d\theta_{t}^{I}}{dt}=\beta \theta_{t}^{S}\theta_{t}^{I}-\gamma \theta_{t}^{I},\frac{d\theta_{t}^{R}}{dt}=\;\gamma \theta_{t}^{I} \end{array}$$

By the fourth order Runge-Kutta (RK4) approximation:

$$\begin{array}{l} f\left(\theta_{t-1},\beta ,\gamma \right)=\left(\begin{array}{l} \theta_{t-1}^{S}+1/6\left[k_{t-1}^{S_{1}}+2k_{t-1}^{S_{2}}+2k_{t-1}^{S_{3}}+k_{t-1}^{S_{4}}\right]\\ \theta_{t-1}^{I}+1/6\left[k_{t-1}^{I_{1}}+2k_{t-1}^{I_{2}}+2k_{t-1}^{I_{3}}+k_{t-1}^{I_{4}}\right]\\ \theta_{t-1}^{R}+1/6\left[k_{t-1}^{R_{1}}+2k_{t-1}^{R_{2}}+2k_{t-1}^{R_{3}}+k_{t-1}^{R_{4}}\right] \end{array}\right). \end{array}$$

#### Extended SIR Model With Time-Varying Transmission Rates

The transmission rate is constant in the SIR model. It should be noted that in actual situations, the speed of transmission can be changed through many interventions, such as personal protective measures, community-level isolation, and city blockade. As is shown below, the eSIR model adds transmission modifier π(t) to the SIR model, so it allows a time-varying probability of the transmission rate.


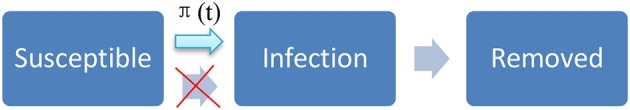


$$\begin{array}{l} \frac{d\theta_{t}^{S}}{dt}=-\beta \pi \left(t\right)\theta_{t}^{S}\theta_{t}^{I},\frac{d\theta_{t}^{I}}{dt}=\beta \pi \left(t\right)\theta_{t}^{S}\theta_{t}^{I}-\gamma \theta_{t}^{I},\frac{d\theta_{t}^{R}}{dt}=\;\gamma \theta_{t}^{I} \end{array}$$

Technically, the RK's approximate of *f* function may be easily obtained by replacing β by β π(t).

#### Markov Chain Monte Carlo Algorithm

We implemented the MCMC algorithm to obtain posterior estimates and credible intervals of the unknown parameters in the above models, including R0, β, and γ. The prior distributions are specified according to the SARS data from Hong Kong as follows (13):

$\theta_{0}\sim \text{Dirichlet}\left(1-Y_{1}^{I}-Y_{1}^{R},Y_{1}^{I},Y_{1}^{R}\right);$
*R*_0_ ~ Log N(1.099, 0.096) with E(*R*_0_) = 3.15, *SD*(*R*_0_) = 1;
γ ~ Log N(−2.995, 0.910) with E(γ) = 0.0117, *SD*(γ) = 0.1, β = *R*_0_γ;
κ ~ Gamma(2, 0.0001), λ^*I*^ ~ Gamma(2, 0.0001), λ^*R*^ ~ Gamma(2, 0.0001).

#### R Software Package

We carried out our predictions with an R software package—eSIR which can output the Markov Chain Monte Carlo (MCMC) estimation, inference, and prediction. The model can also yield the turning points of the epidemiological trend of COVID-19. The first turning point was defined as the mean predicted time when the daily proportion of infected cases becomes smaller than the previous ones. The second turning point was defined as the mean predicted time when the daily proportion of removed cases (i.e., both recovered and dead) becomes larger than that of infected cases. An end point was defined as the time when the median proportion of current infected cases turn to zero. All figures are plotted by the eSIR package.

The transmission rate modifier π(t) can be specified according to actual interventions in different times and regions. According to Chinese government isolation measures and previous study, we set π(t) = 0.9 if t ∈ (Jan 23, Feb 04), city blockade; π(t) = 0.5 if t ∈ (Feb 4, Feb 8), enhanced quarantine; π(t) = 0.1 if t > Feb 8, more enhanced quarantine in Hunan. In the opinion of the Italian government isolation measures, we set π(t) = 0.95 if t < Mar 10, some cities blockade; π(t) = 0.9 if t ∈ (Mar 10, Mar 22), country blockade; π(t) = 0.5 if t ∈ (Mar 22, Mar 31), shutdown of all non-essential businesses and industries; π(t) = 0.1 if t >Mar 31, more international aid and enhanced quarantine in Italy.

We did all analyses in R (version 3.6.2).

## Results

### Epidemic Development of COVID-19 in Italy Compared With Hunan

**Figure 1:**
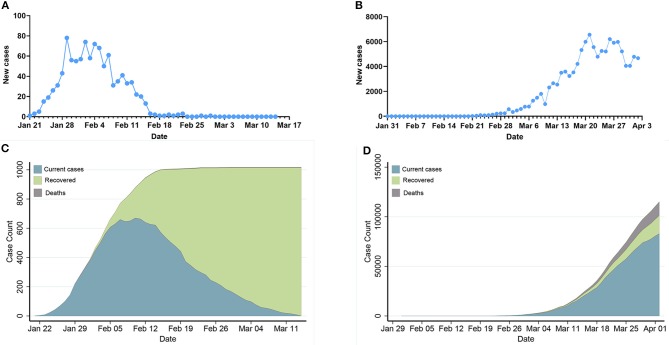
Epidemic development of COVID-19 in Hunan, China and Italy. **(A,B)**: Daily new COVID-19 cases in Hunan, China and Italy. **(C,D)**: Epidemic distribution of COVID-19 in Hunan, China and Italy.

Figure 1 demonstrates daily new COVID-19 cases and epidemic distribution of COVID-19 in Hunan, China and Italy. The number of new cases and confirmed cases show an exponential trend since Feb 21 in Italy while the number of new cases turns to zero from Feb 29 in Hunan.

### Prediction of the Epidemics Trend of COVID-19 in Italy Compared With Hunan

**Figure 2:**
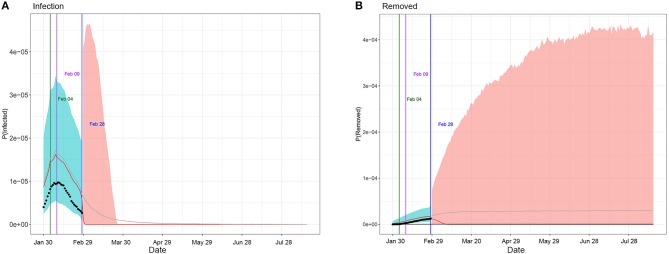
Epidemiological trend of COVID-19 under existing preventions in Hunan, China according to eSIR model. The black dots left to the blue vertical line denote the observed proportions of the infected and removed compartments on the last date of available observations or before. The blue vertical line denotes time t_0_. The green and purple vertical lines denote the first and second turning points, respectively. The cyan and salmon color area denotes the 95% credible interval of the predicted proportions of the infected and removed cases before and after t_0_, respectively. The gray and red curves are the posterior mean and median curves. **(A)** Prediction of the infection of COVID-19; **(B)** prediction of the removed of COVID-19.

**Figure 3:**
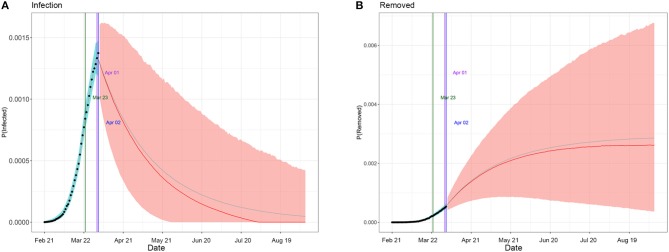
Epidemiological trend of COVID-19 under existing preventions in Italy according to eSIR model. The black dots left to the blue vertical line denote the observed proportions of the infected and removed compartments on the last date of available observations or before. The blue vertical line denotes time t_0_. The green and purple vertical lines denote the first and second turning points, respectively. The cyan and salmon color area denotes the 95% credible interval of the predicted proportions of the infected and removed cases before and after t_0_, respectively. The gray and red curves are the posterior mean and median curves. **(A)** Prediction of the infection of COVID-19; **(B)** prediction of the removed of COVID-19.

Table 1 summarizes the posterior values of R0 and endpoint in Hunan and Italy according to SIR and eSIR model. There would be a total of 3 369 infected cases (95%CI:840–8 013) in Hunan. There would be a total of 182 051 infected cases (95%CI:116 114–274 378) under the current country blockade in Italy. Based on the eSIR model, Figures 2, 3, respectively, indicate an epidemiological trend of COVID-19 under existing preventions in Hunan, China and Italy. The first and second turning point in Hunan appeared on Feb 04 and Feb 09. The first and second turning point in Italy is Mar 23 and Apr 01. The predictions suggest that the endpoints of the COVID-19 epidemics in Hunan and Italy will come on Mar 3 (95%CI: Feb 29 to Mar 28) and Aug 05 (95%CI: May 30 to Inf), separately. Based on the SIR model, Figures S1, S2, respectively, indicate an epidemiological trend of COVID-19 under existing preventions in Hunan, China and Italy (see Supplementary Material).

**Table 1:** R0 and endpoint in Hunan and Italy according to SIR and eSIR model.

	**R0**			**Endpoint**	
**Model**	**Median**	**Mean**	**95%CI**	**Mean**	**95%CI**
**SIR**					
Hunan	2.48	2.58	1.48–4.29	2020/5/17	2020/3/1-Inf
Italy	3.03	3.10	2.14–4.42	Inf	Inf-Inf
**eSIR**					
Hunan	3.05	3.16	1.73–5.25	2020/3/3	2020/2/29-2020/3/28
Italy	4.27	4.34	3.04–6.00	2020/8/5	2020/5/30-Inf

*Inf: The endpoint appears more than 200 days after t_0_*.

## Discussion

This impact of the COVID-19 response (overall quarantine regulations, social distancing, and isolation of infections) in China is encouraging for many other countries (14). We compared the situation in Hunan, China, which has a similar population to Italy to calculate our predictions. The spread of COVID-19 in Hunan Province appeared relatively early and has now entered a phase of no infections, which helps to observe the entire course of the epidemic. Due to the similarity of population size and geographical location adjacent to Hubei, Hunan's public health measures can provide useful guidance for Italy in preventing the further spread of COVID-19.

In our study, the eSIR model with R software package was used to evaluate the impact of intervention measures on the Italian COVID-19 epidemic. In previous studies, estimation of the epidemic of an infectious disease is often performed using constant parameters (15–18). The advantage of the eSIR model is that it combines time-varying isolation measures and expands the SIR model to adapt to the time-varying transmission rate in the population. Lili Wang et al. found that COVID-19 outside Hubei in China has been, so far, much less severe (9). But they did not perform each province's analyses. The first and second points in our study are, respectively, Feb 04 and Feb 09,which are the same as these outside Hubei in China. Furthermore, the actual number of infected cases (1,018) is included in the predicted number of infected cases (840–8 013) and the endpoint (Mar 14) is included in the predicted endpoint (Feb 29 to Mar 28) in our study, which also reflects the stability and accuracy of the eSIR model. Combining the above data and methods, these findings show that the eSIR model is more suitable for predicting the epidemic trend of COVID-19.

Li Qun et al. estimated R0 to be 2.2 (95% CI, 2.09–6.02) among the first 425 patients in Wuhan, China (19). Other studies estimated R0 to be 1.4–2.5 (20), 2.68 (95% CI 2.47–2.68) (3), 3.6–3.8 (21), and 6.47 (95% CI 5.71–7.23) (7). Ying Liu et al. found that the estimated mean R0 for COVID-19 is around 3.28, with a median of 2.79 and IQR of 1.16 by reviewing R0 of COVID-19 in 12 studies (22). Our results showed that the mean of R0 was estimated to be 2.58 (95% CI, 1.48–4.29) and 3.16 (95% CI, 1.73–5.25) in the SIR model and eSIR model in Hunan. which is in agreement with these findings. But our results showed that the mean of R0 was estimated to be 3.10 (95% CI, 2.14–4.42) and 4.34 (95% CI, 3.04–6.00) in the SIR model and eSIR model, respectively, in Italy, which is larger than that in Hunan. Cosimo Distante et al. found that many regions in Italy reach an R0 value of up to 4, some even reaching 5.07 (23), which is similar to our study. This needs to be confirmed by further studies. It is worth pointing out that the estimation R0 in the eSIR model is larger than those in the SIR model. This is because the estimation R0 in the eSIR model is adjusted according to the effect of intervention.

This study showed that COVID-19 spread rapidly throughout Italy after Feb 21. Possible reasons for such rapid growth of infections include: (1) more timely caution and preventative measures were not taken, and (2) the number of infections during Jan 31-Feb 20 could be under-reported due to underdiagnosis, given subclinical or asymptomatic cases. The incubation period for COVID-19 is thought to be within 14 days following exposure, with most cases occurring ~4–5 days after exposure (19, 24, 25). So it seems impossible to for there to have been a total of only two or three cases during Jan 31-Feb 20 in Italy. In addition, the rapid increase in the number of infections after Feb 21 might reflect a belated realization of the spread of COVID-19.

Previous studies have shown that more rigorous government control policies were associated with a slower increase in the infected population (6, 17, 26–29). In our study, compared with no intervention in the SIR model (Figures S1, S2), rigorous government control policies in Hunan and Italy dramatically decreased the number of COVID-19 cases. Based on our model, Italy should still maintain all levels of quarantines as China did by Aug 05 (95%CI: May 30 to Inf). Furthermore, Tianyi Qiu et al. found that delaying the lockdown by 1–6 days in Wuhan would expand the infection scale 1.23–4.94 times and the epidemic would be out of control if lockdown had been imposed 7 days later (18). Our study also shows that taking government control earlier can decrease the number of infected cases by comparing the epidemic trend in Hunan and Italy. In addition, from China's experience, various control measures, including the early detection and isolation of individuals with symptoms, traffic restrictions, medical tracking, and entry or exit screening, can well-prevent the further spread of COVID-19. These measures are in line with the latest recommendations by the World Health Organization and a previous study in Spain (30). But the most effective strategy still needs to be confirmed by further studies. Consequently, it is better and necessary to apply strict public health measures in other European countries with a high number of COVID-19 cases.

Our study has some limitations. Firstly, due to the finite number of tests performed, the asymptomatic and unconfirmed cases may be ignored, and the real number of infected people in Italy, as in other countries, is estimated to be higher than the official count. Secondly, incubation period was not considered in this study. Khalid Hattaf et al. found if time delay or incubation period is ignored, R0 in a delayed SIR model would be overestimated (31). The eSIR model can be further extended by incorporating the incubation period for accurate predictions. Thirdly, since the suspected cases and the daily number of hospitalized cases are not available, they have not been considered in the eSIR model. Fourth, some unforeseeable factors may affect these estimated data in our study, such as the existence of super-spreaders.

In conclusion, the current study is the first to provide a prediction for an epidemic trend after strict prevention and control measures were implemented in Italy. Our study suggests that rigorous measures like China should still be maintained in Italy by Aug 05 to prevent further spread of COVID-19.

## Data Availability Statement

Publicly available datasets were analyzed in this study. This data can be found here: https://github.com/CSSEGISandData/COVID-19.

## Author Contributions

JW, HK, LM, and HY contributed to the study design. JW, HK, and LM contributed to the writing of the manuscript. JW, HK, SY, and CW contributed to the data analysis. SY, CW, and WJ contributed to the data compilation. WJ, YS, WS, and HY contributed to critical review. TP, KF, and LJ contributed to the literature search. YS, WJ, and KF contributed to the design of tables and figures.

## Conflict of Interest

The authors declare that the research was conducted in the absence of any commercial or financial relationships that could be construed as a potential conflict of interest.
