# Supplementary material for: Extended SIR Prediction of the Epidemics Trend of COVID-19 in Italy and Compared With Hunan, China
Source: Front Med (Lausanne). 2020 May 6;7:169. doi: 10.3389/fmed.2020.00169 (PMC7218168; doi:10.3389/fmed.2020.00169)
Supplement: Supplementary file 1 [file Data_Sheet_1.docx]

**Supplementary material**

**Figure S1. Epidemiological trend of COVID-19 under no preventions in in Hunan, China according to SIR model.** **(A): Prediction of the infection of COVID-19; (B): Prediction of the removed of COVID-19.**


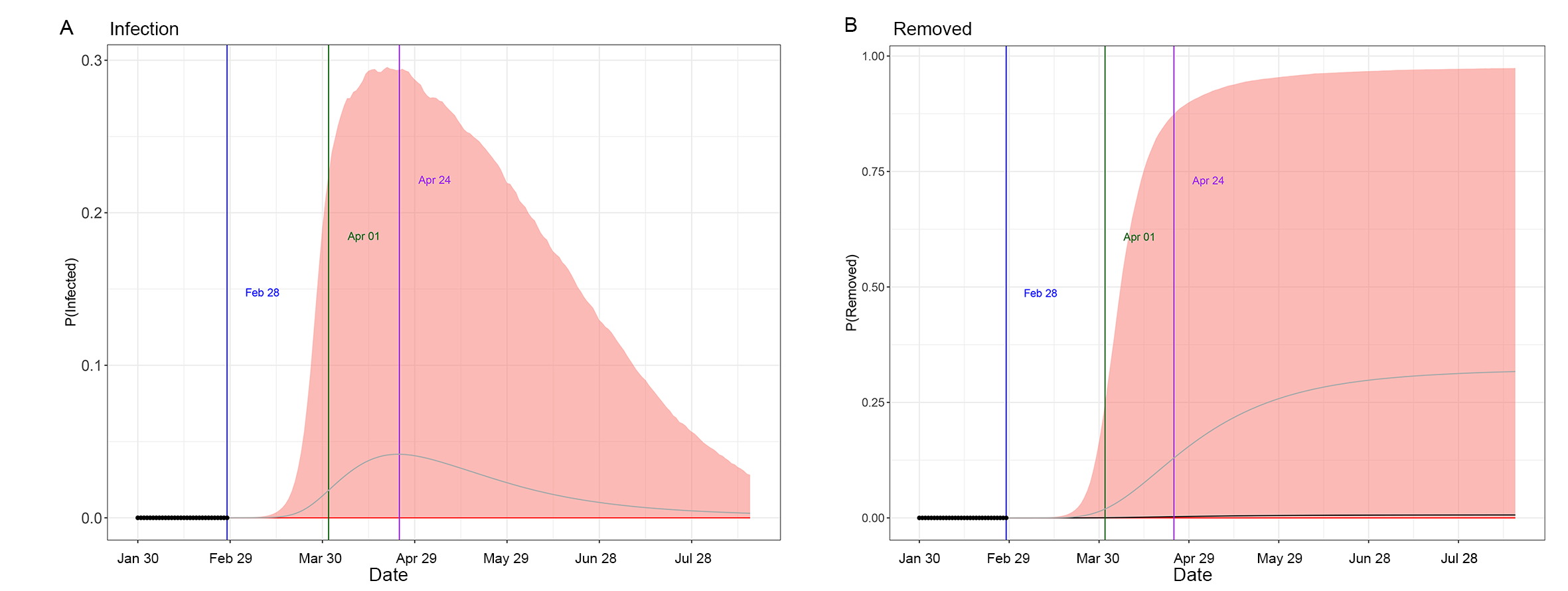


The black dots left to the blue vertical line denote the observed proportions of the infected and removed compartments on the last date of available observations or before. The blue vertical line denotes time t0. The green and purple vertical lines denote the first and second turning points, respectively. The cyan and salmon color area denotes the 95% credible interval of the predicted proportions of the infected and removed cases before and after t0, respectively. The gray and red curves are the posterior mean and median curves.

**Figure S2. Epidemiological trend of COVID-19 under no preventions in Italy according to SIR model. (A): Prediction of the infection of COVID-19; (B): Prediction of the removed of COVID-19.**


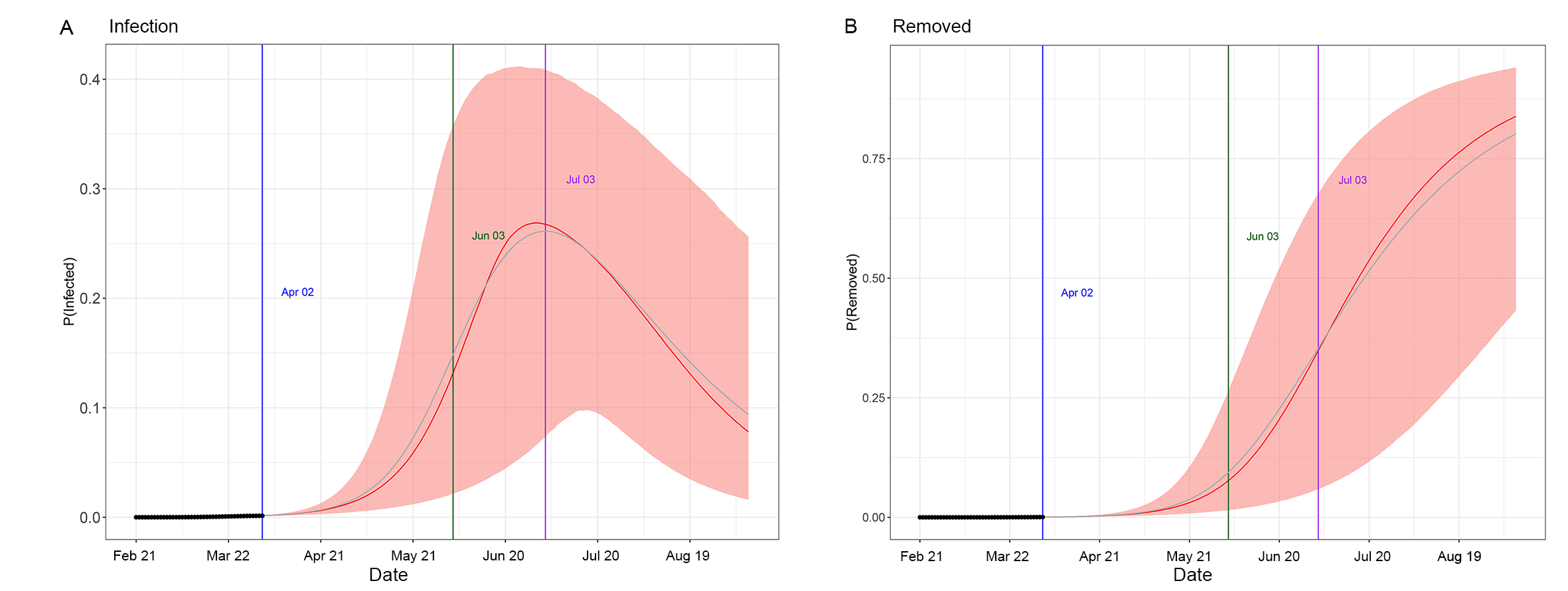


The black dots left to the blue vertical line denote the observed proportions of the infected and removed compartments on the last date of available observations or before. The blue vertical line denotes time t0. The green and purple vertical lines denote the first and second turning points, respectively. The cyan and salmon color area denotes the 95% credible interval of the predicted proportions of the infected and removed cases before and after t0, respectively. The gray and red curves are the posterior mean and median curves.
